# Understanding the Web of Life: The Birds, the Bees, and Sex with Aliens

**DOI:** 10.1371/journal.pbio.0060047

**Published:** 2008-02-26

**Authors:** Jason M Tylianakis

## Abstract

Interactions in food webs indicate the structure and stability of ecosystems. Now, new research uses these interactions to illustrate the vulnerability of pollination webs to invasive plants and pollinators.

When I was in school, I learned about a linear food chain in which, for example, flowers provide food for bees, which in turn are eaten by birds. The implications of this model are clear: if bees were to vanish, birds would starve and flowers would not be pollinated. Whether this concept was a hangover from the old idea of the great chain of being (*scala naturæ*) or a simplification deemed necessary for unsophisticated school children is unclear. Nevertheless, despite the appeal of this simple caricature, it couldn't be further from the truth in most of the world's ecosystems. Birds feed on a variety of plants and animals, and are themselves fed upon by mammals, other birds, a diverse array of parasites, and eventually carrion feeders. It takes little more than a passing glance at the natural world to notice that complexity is the rule, rather than the exception. Describing this complexity and understanding its importance, however, is anything but simple. Ecologists have for a long time struggled to find consistent patterns in the structure of complex webs of interacting species from disparate ecosystems. However, recent empirical and theoretical breakthroughs have begun to shed light on the structure of the web of life that connects living things, and the vulnerability of this web to perturbations such as the introduction of invasive alien species.

## The Structure of Interaction Webs

**Figure 1:**
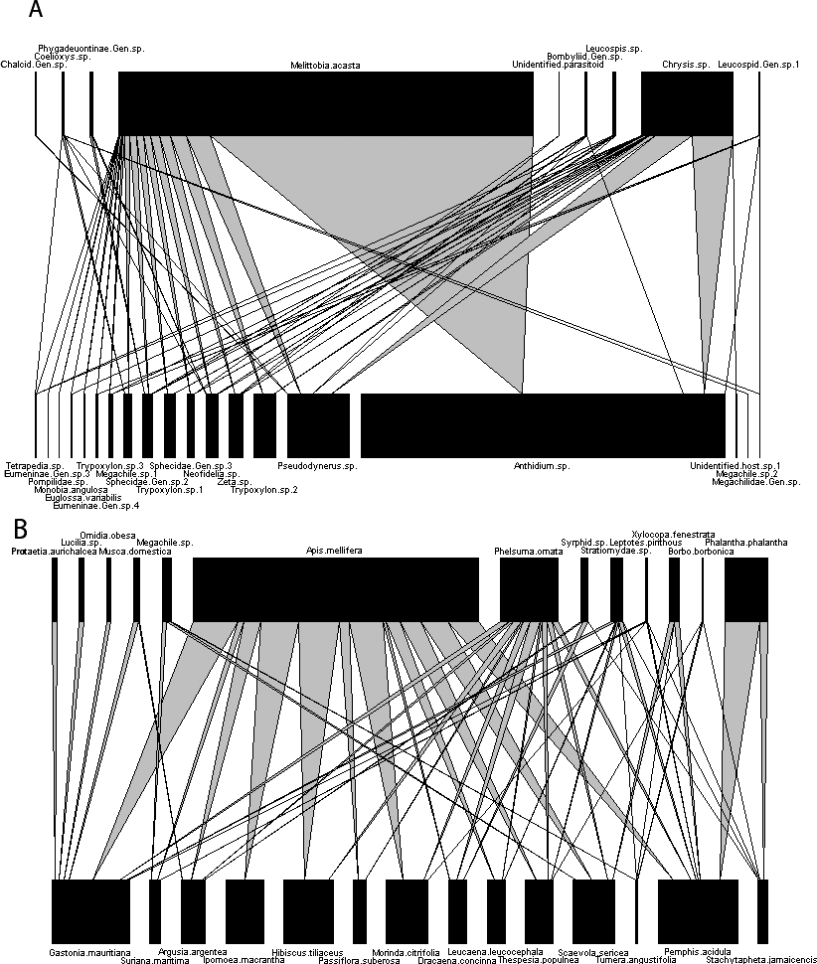
Examples of Quantitative Interaction Webs (A) An antagonist web of bees and wasps (hosts) and their natural enemies (all sites of [6] combined). See Video S1. (B) A mutualist web of pollinators and plants (the Aigrettes web described in [17], which was included in the analysis of [12]). See Video S2.

Interaction webs have a long pedigree in ecological research. For decades, theoretical work has linked the structure of food webs to their ability to resist perturbations [1–3]. In particular, the proportion of weak versus strong interactions within a web can determine its resistance to the extinction of particular species [4]. In these cases, “interaction strength” is measured roughly as the proportion of individuals of a species at the lower trophic level fed upon by, or interacting with, a species at the higher trophic level. Despite the solid grounding and importance of this concept in theory, quantifying interaction strength in diverse, real-world webs presents practical hurdles, and a major breakthrough in empirical food web research was the quantification and graphical representation of community-scale interaction webs (e.g., [5]) (Figure 1). This work paved the way for research examining changes to the structure of real-world biological communities brought about by human activities. Metrics that describe interaction structure can allow us to detect subtle shifts in entire communities of organisms better than coarse metrics such as diversity (the number of species and their relative abundance). This is because interaction structure is vulnerable to the presence, identity, phenology, physiology, behavior, and diversity of different species, so interactions are likely to show changes before a loss of diversity becomes apparent. For example, it was recently shown that the entire structure of antagonistic webs involving bees, wasps, and their natural enemies could be altered by human land use practices, even though there was no apparent change in species diversity [6]. This kind of replicated web analysis provides a link between our theoretical understanding of the importance of interaction structure and the applied necessity of measuring ecosystem change following human activities. It has even been suggested that the value of conserving interaction structure has been overlooked by the traditional focus on simple measures such as biodiversity [7].

## Linking Webs to Ecosystem Services

Interaction webs allow us to visualize the structure of entire communities of species and the ways in which they depend on one another. Not only is this structure critical for the stability of ecosystems (see for example [3]), but many of the interactions that comprise these webs provide services upon which human well-being is dependent. Parasitoid–host food webs (Figure 1A) have an obvious link to biological pest control, and pollinator–plant mutualisms (Figure 1B) are critical for the fruit, vegetable, or seed production of three-quarters of the leading global food crops [8]. Many mutualisms between plants and pollinators are tightly coevolved, with plants depending on pollinators for sexual reproduction, and pollinators depending on plants for floral food resources.

Adequate pollination of a variety of plants often requires a diverse pollinator assemblage [9], yet global loss of natural habitats and agricultural intensification have led to a reported decline in pollinator diversity. This decline has raised fears of a “global pollination crisis,” but the importance of reduced pollinator diversity depends on the abilities of other species to “fill the gap” and take on the pollinating role of locally or globally extinct species. In particular, introduced species such as honeybees could in theory compensate for lost native pollinator species, provided that there was no difference in their ability to pollinate the native plants. The specialization of pollinator mouthparts on particular flower types [9] suggests that such compensation may be unlikely, but the overall effects of changes to pollinator communities on plant reproduction can only be assessed by evaluating diverse networks of plant–pollinator mutualisms.

The melding of theoretical and empirical food web ecology has uncovered some interesting generalities across mutualist networks. Jordi Bascompte and colleagues [10] recently compared a variety of plant–animal mutualist networks from different regions. They found that, despite differences in the kind of mutualism (pollination or seed dispersal), geographic location, and identity of the species involved, there were some consistent patterns in the structure of these webs. The interactions within mutualist networks were found to be highly asymmetric, such that when a plant species depends strongly on an animal species (e.g., a seed-dispersing bird), that animal depends little on that plant (it feeds on a variety of species). Bascompte et al. used a dynamical model to show that these interaction asymmetries, the low average degree of dependence among individual pairs of species within the web (“mutualism strength”), and the high heterogeneity in interaction strengths of the different species all promote coexistence of the mutualisms and maintenance of biodiversity.

## Alien Species Invade Mutualist Webs

**Figure 2:**
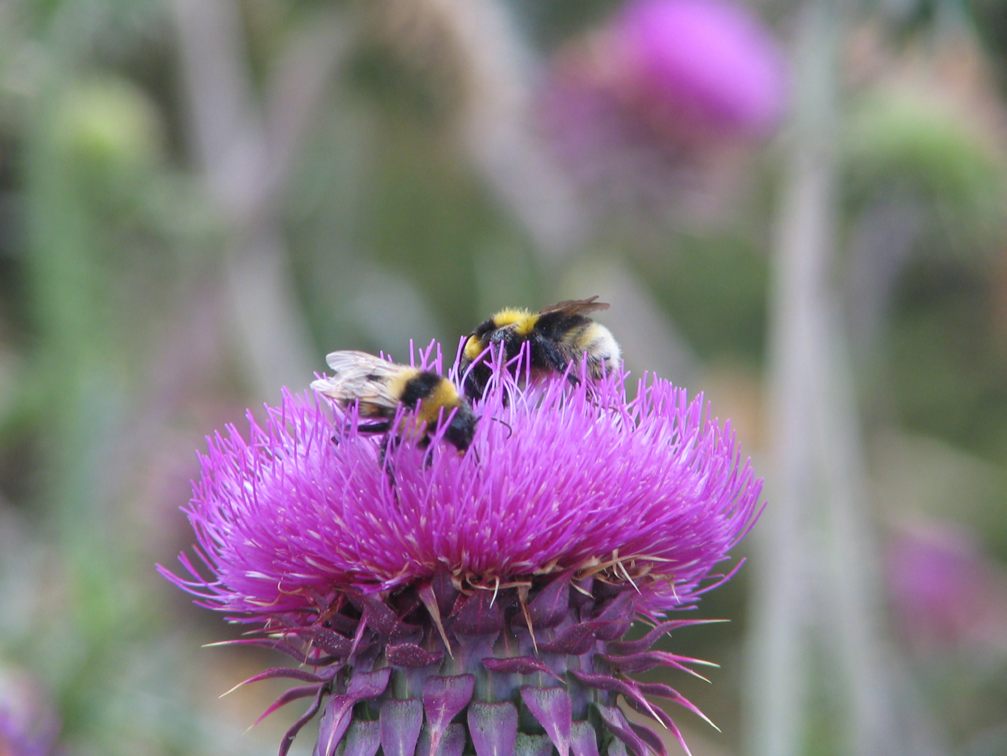
Flower Heads of the Alien Thistle, Carduus thoermeri, Are Visited by Workers of the Invasive Bumble Bee, Bombus ruderatus, in the Temperate Forests of the Southern Andes (Photo: Nestor Vidal)

Invasive species are widely regarded as one of the major drivers of biodiversity loss, due to their frequent dominance of habitats and exclusion of, or predation on, native species [11]. However, in terms of maintaining ecosystem functions such as pollination, provided that the plant gets fertilized, it may be irrelevant whether the pollinating species is native or invasive (Figure 2). This makes the integration of alien species into pollination webs a fascinating natural experiment, as it is not obvious whether invasive species will dominate the interactions within the web, thereby affecting its structure and the ability of native plants and pollinators to interact, or whether they will simply integrate seamlessly into the web and potentially even help native plants to reproduce sexually.

A new study published in *PLoS Biology* by Marcelo Aizen and colleagues [12] in Argentina directly addressed this issue, using replicated webs of plants and their pollinators from islands and mainland to test the effects of alien species on the structure of pollination networks. Their use of diverse, natural assemblages of native and invasive plants and pollinators allowed community-scale structural changes following invasion to be tested in ways that were not previously possible, yielding exciting results. They analyzed ten natural plant–pollinator webs, eight from forests of the southern Andes and two from oceanic islands. These webs were paired geographically, with a highly invaded and a less invaded web comprising each pair. This design allowed them to measure how the strength of interactions and the overall connectivity of the web changed with differing degrees of invasion. They measured the strength of the mutualism using the average frequency of interactions between plants and pollinators, which is strongly related to plant reproductive success and presumably to the nutritional benefit derived by a particular pollinator species.

**Figure 3:**
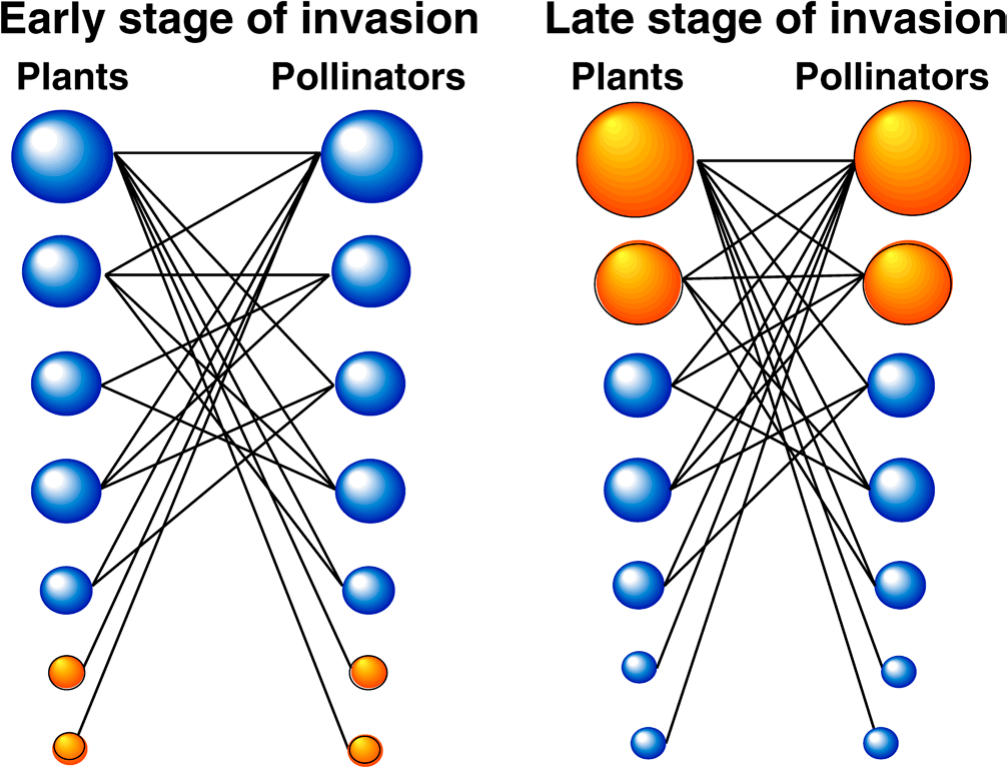
Plant–Pollinator Webs Containing Native (Blue) and Exotic (Orange, Dark Outline) Species, During the Invasion Process Studied by Aizen et al. The size of the circles is proportional to the interaction frequency of a species, which is highly influenced by its abundance. At the beginning of the invasion process (left web), exotic species are found in low abundance, and they interact preferentially with native species. At the end of the invasion process (right web), the exotic species have become highly abundant. Exotic plants become highly attractive by virtue of their abundance, and exotic generalist pollinators are found to forage indiscriminately across plant species. Note that the “super-generalist” exotic species (top right) interact more frequently (have larger circles) than the native generalists (top left). (Figure courtesy of M. Aizen)

For all five pairs of webs they examined, the web with the highest incidence of alien plants also had the highest incidence of alien pollinators, indicating that the same forces may drive invasions by both taxa. They found that exotic species showed low specialization in the species with which they would interact (i.e., they were highly generalist) in the highly invaded webs. Pollination interactions (the “links” between species) were usurped from many generalist native species by the “super-generalist” invaders (Figure 3). This meant that although the connectivity of the network remained unaltered by invasion, the interacting partners changed from native to alien [12]. The mutualism strength of the entire network (the average dependence of each plant on each pollinator and vice versa) declined with increasing levels of invasion. Similarly, in highly invaded webs, there was a high asymmetry in the strength of interactions involving alien mutualists, compared with those interactions involving native species only, and this pattern was not apparent in the lightly invaded webs. This means that aliens engage disproportionately in the most asymmetric interactions as the invasion process progresses.

As mentioned above, interaction strength and the distribution of asymmetries are crucial determinants of the stability of interaction webs [10]. Thus the finding by Aizen et al. that alien species become central nodes and alter these structural attributes of the network, particularly in the advanced stages of invasion, has important implications. Reduced interaction strength and high asymmetry are typically associated with long-term persistence of community structure [10], implying that invader-dominated webs may resist the restoration of interactions among native species to their previous uninvaded state. The core of interactions among super-generalist aliens in highly invaded webs could potentially cause a positive feedback loop, whereby invasive species increasingly enhance their reproductive success and dominance. This would occur at the expense of interactions between native species, as the overall connectivity of the webs did not change.

These changes will expose native mutualists to novel ecological and evolutionary dynamics [12]. Alien invaders, by virtue of their extreme generalism, may cause the fusion of individual modules (subgroups of frequently interacting species), with profound effects on network functioning, reciprocal selection regimes, and the spread of perturbations throughout the web [13]. Furthermore, reduced interaction frequency may be only part of the problem for native species, as researchers from England have recently shown that pollen from invasive species can dominate a web, such that native plants receive invasive pollen from pollinators far more often than they receive their own pollen, which they need for reproduction [14].

## The Future of Interaction Webs

Although quantifying the strength or frequency of interactions within networks can be difficult and time-consuming, it is ultimately the only way in which we can hope to understand the large-scale and often indirect impacts of perturbations to ecological communities. Whereas previous studies have frequently focused on simple food chains or interactions between pairs of species, the results are often dependent on the identity or life history of the chosen species (e.g., whether they are specialists or generalists). Furthermore, propagation of indirect effects throughout the community cannot be measured with such simplified model systems, and burgeoning theoretical and empirical research on interaction structure will provide answers to many key questions. For example, can highly invaded mutualist webs recover lost interactions? Native species may not interact due to behavioral exclusion from exotics, or due to infrequent encounters as a result of their rarity. These kinds of mechanisms may be reversible once the aliens are removed, whereas evolutionary responses to invasive dominance, or inbreeding due to inhibition of reproduction, may have more lasting consequences. It is also unclear what effect reduced mutualism strength and increased asymmetries will have on overall pollination levels or the stability of pollination through time, or how these factors will interact with other environmental perturbations.

Beyond specific benefits such as understanding invasive species, the utility of network scale analyses may extend to interactions at different levels of biological organization. A network framework is already frequently applied to interconnectedness of the proteome and metabolome [15]. Furthermore, there have been recent attempts to unite genetic and ecological research to assess phenotypic effects of genes at community and ecosystem scales [16]. Whereas phenotypes are traditionally viewed as the end product of direct genetic and environmental influences on individual traits, community and ecosystem phenotypes arise from interactions with other species that comprise the community. If indirect genetic effects of keystone species can indeed give rise to variation in community and ecosystem phenotypes [16], then the framework employed by Aizen and colleagues [12] may one day help to elucidate the indirect effects of invasive genes, such as those carried by genetically modified organisms.

Network frameworks, which are increasingly being used to answer specific questions in disparate disciplines, may shed further light on the complex interactions between biological entities. Whether the role of birds and insect pollinators will be interpreted differently under a more holistic network view remains to be seen, but our understanding of indirect effects within communities of organisms will have certainly deepened through this approach.

## Supporting Information

Video S1 Binary (Nonquantitative) Version of Figure 1A
The antagonist web of bees and wasps (hosts) and their natural enemies (all sites of [6] combined).
(412 KB WMV).

Video S2 Binary (Nonquantitative) Version of Figure 1B
A mutualist web of pollinators and plants (the Aigrettes web from [17]).
(537 KB WMV).
